# Dr. Thomson’s Sermon

**Published:** 1852-03

**Authors:** 


*The Study and Practice of Medicine not unfavorable to the cultivation of a Religious Character. A Sermon, preached by request before the Medical Class of the University of Buffalo, Dec.* 28, 1851. By M. La Rue P. Thomson, D. D.

This sermon, by Dr. Thomson, is an able and eloquent vindication of medicine from the charge so often made that it tends to religious skepticism. The class of medical students, before whom it was delivered, testified their appreciation of its merits in soliciting a copy for the press, and Dr. T. has laid the profession under obligations by consenting to its publication. We wish it might be extensively circulated.

